# ‘Demystifying’ the encounter with adolescent patients: a qualitative study on medical students’ experiences and perspectives during training with adolescent simulated patients

**DOI:** 10.1080/10872981.2021.1979445

**Published:** 2021-09-23

**Authors:** Yusuke Leo Takeuchi, Raphaël Bonvin, Anne-Emmanuelle Ambresin

**Affiliations:** a Interdisciplinary Division for Adolescent Health, Lausanne University Hospital (Chuv) and University of Lausanne, Switzerland; b Medical Education Unit, Faculty of Biology and Medicine, University of Lausanne, Switzerland

**Keywords:** Adolescent, simulation, medical education, communication skills, doctor-patient relationship

## Abstract

Training with adolescent simulated patients (ASP) is increasingly recognized as an effective form of teaching interviewing skills with adolescent patients. Beyond the acknowledged effectiveness and satisfaction of training with ASP, little is known on medical students’ actual experience and specific learning needs related to simulated encounters with ASP, as well as factors influencing their learning experience.

The aim of this study was an in-depth exploration of medical students’ perspectives about training with ASP.

Using a qualitative design with grounded theory methods, we conducted in-field observation of training sessions with ASP and individual interviews with eighteen fourth-year medical students participating in training.

When provided with an actual experience in a simulated setting, students go through a process of anticipating then modulating the challenge of the encounter with an adolescent patient. This challenge is influenced and modulated within 3 main dimensions: preconceptions about adolescents, level of experience with adolescent patients and professional distance. This process is also influenced by how students perceive and cope with the educational setting.

Training with ASP, as a first concrete experience of an adolescent consultation, is an opportunity to address important aspects of students’ attitudes towards adolescent patients such as students’ preconceptions, personal experiences and feelings that could influence the doctor-patient relationship later on. Training should focus on ways to reflect upon and handle such attitudes and the emotional resonance experienced by medical students.

## Introduction

Most medical students will have to take care of adolescent patients at some stage during their medical practice. They will face the challenge of identifying major causes of morbidity among adolescents like mental health disorders, injuries or substance use, even though the most common reasons for consultation are various somatic symptoms [1–3]. To meet this challenge and to address sensitive issues like sexual or mental health, physicians need to provide a trustful and confidential environment [4,5]. Adolescents rate their care positively when physicians are friendly, respectful and show appropriate communication skills [1,6].

Adolescent simulated patients (ASP) are young people (adolescents or young adults) trained to portray the role of adolescent patients. Simulation-based training with ASP is increasingly recognized as a way to improve health professionals’ clinical skills with adolescents. Such training is effective in improving interviewing and counseling skills regarding general adolescent health issues [7] and other specific skills like the assessment of adolescent depression and suicide risk [8–10]. The quality of ASP performance and feedback is also highly valued, as reflected in high satisfaction among learners [11–14]. The perceived authenticity of the ASP performance evidenced in previous research [14] is an essential ingredient for a positive experience. In addition, there are no major risks for adolescents hired as simulated patients [15–18].

Beyond the fact that training with ASP is a recognized and effective training method that is highly appreciated by students, little is known on medical students’ actual experience and specific learning needs related to simulated encounters with ASP. For example, it is not clear whether students participating in such training have more needs in the learning domain of clinical skills (what they do) – i.e., interviewing and communication skills – or more in the affective or attitudinal domain (what they feel) – i.e., those associated with thoughts and feelings in the relationship with patients [19]. Yet, an important issue for trainers, especially if training sessions are short or scarce, is to identify on what to focus during such training.

We also do not know to what extent the experience of such training is influenced by some individual features (such as prior representations about adolescents, experience and confidence in the care of adolescents, etc.) or features related to training setting (e.g., group vs individual setting, age & training of simulated patients etc.).

Another question that remains is whether the involvement of a simulated patient is absolutely necessary or whether the same learning outcomes could be achieved with another teaching method. Training with ‘adult’ simulated patients is often valued by students for practicing communication skills, particularly because of its experiential nature [20,21] and also because it allows them to prepare for real patients in a secured setting and to obtain structured feedback [22]. Few studies, however, have focused on students’ experiences with adolescent simulated patients.

In a context where adolescent health is globally poorly represented in undergraduate curricula [23], it is essential to understand students’ needs in order to help trainers and curriculum designers define focused objectives and select the appropriate teaching method to improve the effectiveness of training.

The purpose of this study was an in-depth exploration of medical students’ perspectives regarding training with ASP. Our research questions were: (1) *What are students’ prior conceptions of encounters with adolescents and their needs and expectations in relation to a training session with ASP?* (2) *How do medical students experience their participation in training?* (3) *What do students perceive as the added value of a training session with ASP?*

## Methods

We conducted a qualitative study using grounded theory (GT) methods. The aim of GT is to build an explanatory theory about social processes that emerges from empirical data [24–27]. Fundamentals of GT that were applied to this study include theoretical sampling, the iterative process and use of the constant comparison method during data analysis [25]. Theoretical sampling means that research participants are purposefully selected if they are likely to provide answers, initially to the research questions and later on to the categories emerging from the analysis [25,28]. The iterative process implies that data collection and analysis are performed simultaneously, each informing and influencing the other [25,27]. The constant comparison method means that as coding of data processes, raw data, codes and categories from all sources of data are constantly compared to look for similarities or differences.

### Setting

The study took place at the medical school of the University of Lausanne, Switzerland, which has a 6-year undergraduate curriculum. Clinical teaching begins in the second year only with simulated patients, and then gradually increases since the third year with the beginning of exposure to real patients. Clinical rotations take place in the second half of the 4th year and electives during the whole 6th year. The undergraduate curriculum in adolescent health and medicine includes 13 hours of formal lectures over 6 years and a three-hour training session with ASP during the fourth year, run by faculty members specialized in adolescent health. At this stage of the curriculum, the large majority of students have not had any contact with adolescent patients in a clinical setting yet.

The main purpose of this simulation-based training session is to practice interviewing and communication skills with adolescents. During this session, groups of approximately ten students encounter two ASP in a row. One student interviews the ASP while the others observe. After 5–10 minutes of role-play, the student gives feedback on their feelings and then gets feedback from the ASP, the observing students and the teacher, focused on interviewing techniques, communication skills and attitudes. The feedback is followed by a brief group debriefing on these aspects. Then, another student carries on with the encounter and so on until all students have participated. A group discussion takes place at the end of the session whose purpose is mainly an evaluation of the training session by the students.

ASP at the time of this study were healthy young people between 14 and 21 years old, carefully selected according to their cognitive skills and affective stability, with parental consent. They were trained to portray a clinical vignette of an adolescent patient (e.g., an adolescent with fatigue, substance use, etc.) and to give structured feedback to students.

### Sampling

We recruited our first 11 participants for interviews among fourth-year medical students who volunteered during an information session ten days before each training session. According to a purposeful sampling strategy, we made sure to include students who would participate in different training sessions – i.e., with different trainers and ASP. Right after training sessions, we recruited 7 additional participants who seemed to have particularly liked or disliked the training or who had, during training, raised some thoughts or displayed attitudes or behaviors that seemed to be related to the emerging categories of the data analysis and that prompted further exploration (theoretical sampling) [28]. The sampling process stopped when no new insights emerged from the interviews (data saturation) [28].

### Participants and data collection

Over a three-month period, the main investigator (YLT) conducted 29 individual in-depth interviews of 45–55 minutes each with eighteen students aged 21 to 27 (M = 5; F = 13). Of the 18 students included, 11 participated in two interviews: the first one a few days before the training session and the second one a few days after training. As mentioned above, seven additional students, recruited right after their training session, participated in one interview after training. The interviews with these 7 participants focused more on their experience of the workshop and how it had responded to their prior representations and expectations. Their inclusion in the study particularly helped refine the emerging categories. We used an interview guide designed beforehand and refined it with insights gained from data collection according to the iterative process of GT. Data collection stopped when no new insights emerged from data analysis (data saturation) [28]. Interviews were audio recorded and transcribed verbatim. Alias names were used in transcriptions and in this article to guarantee the anonymity of participants.

**Figure 1.**
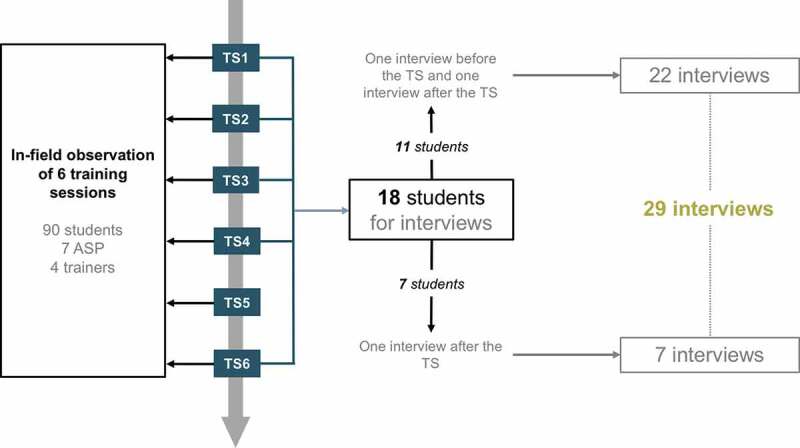
Illustration of data collection and recruitment procedures **–** *TS = training session, ASP = adolescent simulated patients. We conducted in-field observation during training sessions 1 to 6 and recruited 18 students for interviews in training sessions 1 to 4 and 6.*

YLT also conducted in-field observation of six training sessions and collected descriptive (observed events) and reflective (personal thoughts) field notes as a passive observer [29]. YLT had no active role in training sessions. Ninety students, 4 trainers and 7 ASP participated in these 6 sessions. The interviewed students participated in one of the six sessions with in-field observation. Figure 1 illustrates recruitment and data collection procedures.

### Data analysis

The research team (YLT, RB, AEA) performed successive cycles of coding of transcribed interviews and field notes [28]. Initially, all authors read two interviews each and coded them line-by-line (‘initial coding’). They subsequently elaborated a common coding structure that YLT applied to all interviews and field notes (‘focused coding’). Through ‘axial coding’, the authors explored and outlined connections between themes generated during initial and focused coding [25,27,28] and then integrated them around a core category (‘theoretical coding’) to generate theory [30]. At each step of analysis, the research team used memo-writing and applied the GT constant comparison method [25,27]. After analysis, YLT performed member-checking with 5 participants: during a group meeting, he presented the results to them to ensure that they matched with their views [31]. The research team used the online software *Dedoose®* to help organize the analysis.

### Ethics review board approval

The study protocol was submitted to the Ethics Committee for research on human beings of Canton de Vaud, Switzerland, which decided that the need for approval was waived because it did not concern health data. Each participant received an information sheet and written informed consent was obtained from each of them.

## Results

**Figure 2.**
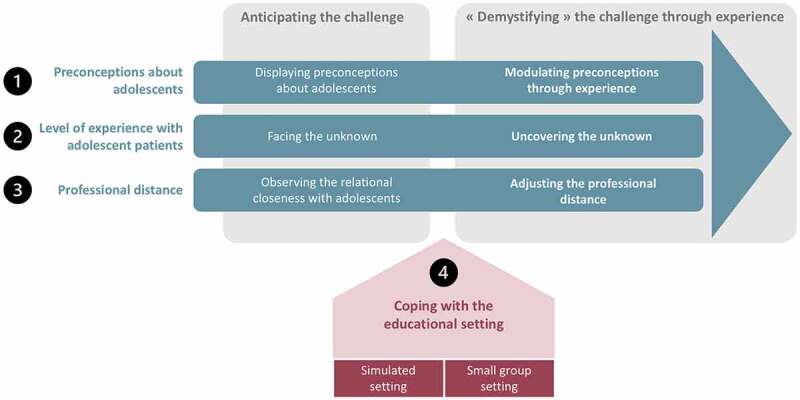
Illustration of the process of anticipating and ‘demystifying’ the challenge through the experience with an adolescent simulated patient

The first phase of analysis *(initial, focused and axial coding)* resulted in the emergence of 3 main categories including 8 themes and 16 sub-themes, as illustrated in Table 1. We then reorganized the themes around the core category ‘Demystifying the challenge through experience’ *(theoretical coding)*. This resulted in the schematization of an experiential process (Figure 2) in which students anticipate and then modulate the challenge of the encounter with adolescent patients through an actual experience of a consultation in a simulated setting.

**Table 1.** Summary of main categories, themes and subthemes that emerged from the first phase of the data analysis (initial, focused and axial coding)

Categories	Themes	Subthemes
**Anticipating the challenge**	Displaying preconceptions about adolescents	Perceived specificities and needs related to the adolescence period Communication & interaction challenges with adolescent patients
	Observing the relational closeness with adolescents	‘Emotional’ closeness with adolescents ‘Social’ closeness with adolescents
	Facing the unknown	Past professional and personal experiences with adolescents Anticipated difficulties with specific skills and knowledge in adolescent health
**‘Demystifying’ the challenge through experience**	Modulating preconceptions through experience	Modulating preconceptions
	Adjusting the professional distance	Dealing with one’s own experience of adolescence Adjusting the relational distance
	Uncovering the unknown	Having ‘done it once’ Observing and practicing concrete interviewing skills
**Coping with the educational setting**	Coping with the small group setting	Fragmentation of the interview Presence of observers Active observation and group discussion
	Coping with the simulated setting	Purpose and benefits of simulation Authenticity and professionalism of ASP

This process of anticipating then ‘demystifying’ the challenge through experience is organized around three main dimensions: *(1) preconceptions about adolescents, (2) level of experience with adolescent patients* and *(3) professional distance*. The process is also influenced by how students perceive and cope with the educational setting. The following paragraphs successively describe how the challenge is influenced and modulated within these 3 dimensions and the role of the educational setting. The appendix provides additional significant quotes and fieldnotes related to these 3 dimensions.

## Preconceptions about adolescent patients

A first important factor that influenced students’ perception of the challenge of interviewing an adolescent patient was their preconceptions about adolescents. This refers to preconceived ideas and opinions about the specificities and needs regarding the phase of adolescence on the one hand, and to interaction and communication with patients of that age on the other.

*‘[I would be afraid to]do something wrong … that could then have huge consequences especially because they are in an unstable period’ (Lauriane, session 4)*

This excerpt illustrates how considering adolescence as a difficult period implies the need for students to take special precautions when interacting with adolescents in order to avoid negative consequences. Moreover, some students expected specific behaviors from adolescents that would make communication and interaction more difficult, *e.g., less ready to talk about themselves, less confident in other people etc.*

*‘Maybe we fear adolescents a bit because we know that it is more difficult to communicate with them, they may be less open about their private life’ (Rachel, session 3).*

Given these considerations, one of the perceived roles of training was to modulate some of their preconceptions and de-dramatize some of the related anticipated difficulties as illustrated in the following excerpt and extract field note:

*‘And the exercise showed me that you shouldn’t generalize. Not all adolescents are completely silent … or intimidated by a white coat.’ (Dimitri, session 2)*

Extract from field notes (session 2): *‘The student says, “It’s not like the other adolescents I’ve seen so far, they would just say yes/no or I don’t know.” The teacher relates her experience by reporting that often adolescents will open up when you know how to get them to talk’*

## Level of experience with adolescent patients

Another factor influencing the level of challenge was the lack of experience of students in adolescent medicine and health. For most of them, this training was the first experience with adolescent patients. While some could rely on some personal experiences outside the medical context, most were not at all familiar with the specificities of adolescent medicine.

*‘ … but in terms of adolescent patients, I had just seen one consultation with an adolescent at the children’s hospital. […] So, it was unfamiliar and [I was] maybe a little bit afraid to talk with adolescents’ (Joachim, session 4)*

At the same time, students identified specific skills required for example to address sensitive topics (sexual health, mental health, suicidal thoughts, substance use, etc.) to set the framework for the consultation or to structure the interview. Most students reported or displayed difficulties related to a lack of such skills, which was also widely noted in the field observations.

*‘Well, I’d feel ok to take care of a baby with diarrhea but I wouldn’t feel ok to take care of an adolescent … who is not feeling well, or running away or self-harming. I’d say: ok we can talk about it but I couldn’t do more […] because I don’t know how to do that’ (Lauriane, session 4).*

Excerpt from field notes (session 4): *“the trainer asks the student to explore sexual health because it’s important in this ASP case. The student answers ironically: ‘Oh great! But I have no idea how to do that!’*

Considering the anticipated challenges described above, many students appreciated the opportunity to just ‘have done it once’ and to have ‘demystified’ the encounter with an adolescent in a medical setting:

‘*It was the first time that I did it with a patient, whether simulated or not. And I think it’s great because it demystifies the whole thing a bit’ (Rachel, session 3)*.

While some students acknowledged that the training was a good opportunity to observe and practice helpful interviewing techniques (e.g., question wording to address sensitive topics or to ensure confidentiality, how to structure the interview, etc.), many highlighted the reassuring role of this first actual experience itself without necessarily identifying specific or structured take home messages.

## Professional distance

Finally, another important challenge was how students managed their sense of relational closeness with adolescents and adjusted their professional distance accordingly. Some could easily recall their own adolescence and potentially identify with adolescent patients on an emotional level:

*‘I rely on my own experience as a teenager. […] I tell myself: ok this kid is potentially in psychosocial distress. Oh and by the way I remember when I was 14, I had this great friend who was depressive and used to cut her arm. I mean, it evokes you that. My past as an adolescent is not that far.’ (Roxanne, session 4)*

Others felt close more on a social level, reporting similar activities or exploratory behaviors (e.g., binge drinking) and some might consider them as potential friends outside the medical setting. This closeness of age challenged their professional role and posture as illustrated in the following excerpt:

*‘I was there not long ago […], so it is easy to identify with them. So, you’re here, facing a young person … and we still need to have the status of a responsible adult. […] I would say that the definition of roles is more complicated. […] I think it’s easier with old people. […] With young adults, not only with adolescents, it’s difficult because outside the frame of the doctor-patient relationship, they could have been a friend.’ (Sarah, session 4)*

Thus, through the role-play, students faced the challenge to adjust the professional distance in the way they manage the clinical situation and interact with patients.

Some students recognized that this proximity could ease the relationship:

‘*We are more able to identify with them, to understand them and be sympathetic’ (Dimitri, session 2)*.

Most students were also very clear about the need not to treat adolescents as friends and also to step back from their own experiences:

*« I think that it’s a mistake to identify too much with patients. And I think that there was a risk here, I mean … Who has never been drunk to the point of throwing up? […] So in the balance, I left out my own experiences as an adolescent and instead I thought: what would a health professional do with that?” (Roxanne, session 4)*

However, a few students displayed difficulty distancing themselves from their own experiences. Some were tempted to share their own experiences with patients or would let these experiences influence their investigation or their interpretation of a situation …

*‘I did the part of history about alcohol and I told her: yeah, you know we all have drunk at some point … to avoid judgment, you see. Then maybe I could get closer to this patient’ (Mathias, session 1)*

… while some were tempted to talk with patients like with friends

‘He (another student) said, did you f*** her? And there was a silence in the room, I think he also realized that this was not the right way to say it. And at the end of the interview, the teacher said that it was not very appropriate, it was not the right way to … to ask the question’ (Lucas, session 1)

## Coping with the educational setting

Several factors regarding training design and delivery, particularly the small group setting as well as the simulation setting, had also a potential influence on the process.

Regarding the small group setting, students needed to overcome some drawbacks. The first one was the fragmentation of the interview, that is to say that each student conducted only a part of the interview:

*‘Taking over the role of someone else, […] it breaks dialogue [and] conversation. And we don’t know really where to start. It becomes unrealistic. Doing it from the beginning to the end … it’s closer to reality.’ (Alec, session 2)*

The second drawback was the need to overcome the stress generated by the presence of observers.

Yet, students also recognized the added value of the small group setting in such training. The active observation of peers was considered a major asset to provide inspiring ideas related to interviewing skills–e.g., how to address sensitive topics:

*‘I remember a [student] who took the sexual history. She asked really good questions and maybe […], I will memorize some of them and keep them for the next [interview].’ (Soraya, session 3)*

Students also underlined that the presence of peers fostered discussion, reflection and experience-sharing positively.

Regarding the simulated setting, one important issue in determining a positive training experience was the perceived authenticity of the encounter and more particularly of ASP because they were real adolescents. Many students appreciated the realism of ASP especially if they attributed their reactions not to a standardized role but to their nature of real adolescents:

*‘[…] she was a real adolescent, unless she was really good in theater. In my opinion, she was a real adolescent who was really waking up and didn’t want to talk.’ (Roxanne, session 4)*

Students also emphasized the role of simulation in providing an opportunity to prepare for real patients in a secured setting, with the possibility to make some mistakes without consequences.

## Discussion

Students’ experiences during training with ASP involved aspects related to attitudes, in addition to and complementary to aspects related to interviewing and communication skills. Our results highlight that training sessions with ASP are an opportunity for students to reveal and explore important issues relating to their attitudes towards adolescent patients such as their representations, personal experiences, feelings and their interpersonal distance with adolescents. Identifying and reflecting upon these issues is crucial because it might strongly influence their relationship with adolescent patients. Indeed, health professionals’ perceptions and experiences are known to influence their beliefs, attitudes and so potentially their care for adolescents [32,33]. In addition, this focus on more subjective and affective aspects is interesting because communication teaching focused solely on ‘tasks’ or ‘skills’ has been criticized in some fields such as cancer care because it does not sufficiently take into account the fact that communication is subjective, depending on relational aspects and on patient perceptions [34]. Alternative approaches focusing on students’ feelings, thoughts and emotions towards patients have thus been developed to teach communication [35].

Students anticipated challenges in their relationship with adolescents depending on their own representations and past experiences. The lack of ease with adolescents has been reported among health professionals who often disclose discomfort and a lack of confidence regarding adolescent health issues [36,37]. Training with ASP is an opportunity to explore and overcome such feelings through this concrete experience. In previous work, following training with ASP, students reported greater confidence with adolescent patients [38]. Lowering students’ apprehension in care of adolescent patients through ASP training is a sound answer to improve the quality of care for adolescents. The feeling of self-efficacy, defined as people’s belief in their ability to perform a task [39], is essential as it is associated with better screening practice for risky behaviors among adolescents [40].

Another interesting finding is that the specificity of training programs with ASP allows medical students to experience the influence of their own past experience as adolescents and of the small age gap on the interpersonal distance with patients. Subsequently, such training represents an opportunity to experience how to fine-tune their professional attitude and posture as well as the framework and limits of the doctor-patient relationship. How professionals adjust the relational distance with adolescent patients is essential because adolescents expect their doctor to be friendly [6], which means someone engaged and interested in their lives, someone they can feel close to and they can easily talk with, yet who is still professional in the sense that they do not try to behave as a friend or adopt an unauthentic communication style to get closer. [6,41,42]. While taking their own experiences into account might promote empathy and a friendly attitude, students need to distinguish *friendly* from *being friends* and to learn to step back from their own experiences and values to find the proper distance with patients [32]. This question of the right professional distance in a sense of maintaining boundaries while being empathetic has been a preoccupation of healthcare professionals for a long time [43,44].

Overall, students appreciated to have ‘done it at least once’, to decrease the perceived challenge, to realize what it means to face an adolescent patient and to help reflect upon and modulate some of their attitudes. The experience itself was thus particularly valued. This suggests the importance of having a concrete practical experience as a trigger for feedback and reflection, which is in line with theories of experiential learning (learning through an experience) [45]. The simulation setting is particularly appropriate because it allows for safe practice before having real patients, structured feedback from the patient and time for reflective practice [22]. Reflective practice is crucial in promoting active learning through an experience [46] and is also an essential ingredient for self-regulated learning and lifelong learning [47]. Such training might thus represent an opportunity among others to support the medical students’ development of reflective practice about their attitudes towards adolescents and advocate for the continuation of such practice in their subsequent care of adolescents, whether supervised or not.

Regarding the educational setting, students highlighted the contribution of a group debriefing in sharing and reflecting on experiences and feelings, which is a well-known benefit of small group learning [48]. Furthermore, the observation of how peers lead the interview could provide a model on how to interview an adolescent patient and promote observational learning [49,50, 51]. However, students regretted the insufficient amount of individual time of simulation and the stress related to the presence of observers. Minimizing these negative feelings is important to avoid their impact on the goals and course of training [35]. As for aspects related to simulation, having real adolescents and young people hired as ASP allowed a more authentic experience and was widely appreciated, in line with previous work [14]. This point is particularly important in a training focused on relational aspects.

### Implications for practice

Our results suggest giving a greater focus on attitudes (affective domain of learning) to provide a sound answer to students’ needs in the field of adolescent health, which has implications for both facilitators of such sessions with ASP and curriculum designers. In terms of facilitation of these sessions, trainers might lead the discussion following the role play in three steps. First, the student should be asked to disclose how they felt during the role play particularly in their relationship with the patient, what difficulties were encountered and why. Second, a concise feedback focused on interpersonal and emotional aspects should be provided, first and foremost by the ASP and supported by the teacher. Third, by promoting group discussion while referring to their concrete experience of the simulated encounter, the teacher needs to actively help students reflect upon their preconceptions and own experiences and how they influence their interaction and professional distance with adolescents. Since students come to the workshop with their own representations, it might be interesting, if time permits, to explore them even before the role play, for example through a group discussion, and to further reflect on the evolution of these representations through the experience with ASP.

As for the educational setting, the small group setting still seems most appropriate for this type of training. The main issue however is to ensure a balance between individual role-play time and group discussion time. Obviously, if resources permit, each student should lead a full interview or at least larger segments of the interview. One possibility would be to reduce the size of the groups (3–5 students) or raise the length of training. Another option would be to prepare students in advance by providing them with a model of some interviewing and communication techniques with adolescents, e.g., a video of a consultation illustrating how to set the framework of the consultation and how to structure the psychosocial history.

Since the question of attitudes and communication skills with adolescents is transversally important whether one is a pediatrician, a general practitioner or another specialist and has specificities compared with adults or children, its integration as an essential component of adolescent health undergraduate curricula is important beyond specific theoretical knowledge. It implies for curriculum designers to manage a space for the expression of and reflection upon these fundamental attitudes, if possible based on a practical experience as a trigger. The implementation of sessions with ASP appears to be an appropriate method in this perspective. In a context where resources do not allow the participation of simulated patients, a small group workshop stimulating reflective practice for example around clinical vignettes, could be an alternative knowing that it would not replace the contribution of experience.

Finally, simulated patients should be recruited among young people matching the age of the role. Their role and especially their way of behaving in the relationship or of reacting to questions should not be standardized in order to maintain a high level of authenticity. Particular attention to feedback on relational aspects should be given during the training of ASP.

### Limitations

This study has several limitations. First, the results cannot be generalized without further research because of the qualitative sampling strategy and the importance of the teaching context and culture (e.g., structure of the teaching curriculum, previous experiences of simulation, assessment approaches, etc.). Secondly, no students from session no.5 volunteered for participation, and three students who were approached by YLT finally declined to participate in an interview. In relation to this, one strength of our study is that we integrated insights from two sources of data according to the *triangulation process* [27,51].

Finally, our involvement in training with ASP or adult simulated patients may have played a role in the analysis [28]. To avoid the influence of our preconceived ideas about training with ASP on data, we constantly reflected on them to take them into account [28] and ensured through member-checking that our analysis was aligned with participants’ points of view [31]. Moreover, as a way to promote this reflectivity and to enrich the analytic process, the entire research team met repeatedly to discuss emerging concepts.

## Conclusion

Our study suggests that practical training integrating simulated patients is essential for undergraduate teaching in adolescent health because it provides a unique opportunity to have at least one experience in a secure environment before real patient encounters. Such an experience is crucial to enable a reflective practice that should be focused on attitudes and professional identity as to help students uncover and address their feelings, personal experiences and preconceptions that might influence their relationship with adolescent patients. Regarding the educational setting, the added value of a small group setting and of hiring ‘real’ young people as ASP are important aspects relevant to trainers in adolescent health and medicine who wish to implement or optimize training with ASP. Future research should evaluate the magnitude and sustainability of the impact of such attitudes-focused trainings on the quality of adolescent care, primarily the relational aspects.

## Data Availability

The datasets generated and/or analyzed during the current study are not publicly available because individual privacy could be compromised but are available from the corresponding author on reasonable request.

## List of abbreviations

ASP: adolescent simulated patient(s)

GT: grounded theory

## Appendix

**Appendix**: additional significant quotes and fieldnotes related to the 3 dimensions of the core category ‘Demystifying the challenge through experience’, resulting from the final phase of the analysis (theoretical coding)

**Table:**

Themes and definitions	Additional significant quotes or field notes
1. PRECONCEPTIONS ABOUT ADOLESCENTS	
Displaying preconceptions about adolescents	
Refers to examples of students’ preconceived ideas about adolescence as a unique period of life with specific needs and about certain specific behaviors of adolescents that influence the way they communicate or interact with professionals	*‘In my head, adolescence is a time when anything can happen, and we have the feeling that we need to handle [adolescents] with kid gloves’ (Dimitri, session 2)*. *‘Maybe we fear adolescents a bit because we know that it is more difficult to communicate with them, they may be less open about their private life’ (Rachel, session 3).*
Modulating preconceptions through experience	
Refers to how the training session helped change the students’ perspective on their preconceptions	*‘The training session allowed me to see that even if adolescents didn’t necessarily want to be there for the interview […], it didn’t prevent them from talking or from establishing a trustful relationship.’ (Sylviane, session 1)*
2. LEVEL OF EXPERIENCE WITH ADOLESCENT PATIENTS	
Facing the unknown	
Refers to how students perceive the challenge related to their lack of experience with adolescents, and the subsequent lack of skills and knowledge in specific areas of adolescent health and medicine	*‘Because here we’re kind of thrown into the deep end, like: “ok let’s go, take the history of an adolescent”. So for us, it’s like, well, do we have to do the HEAADSSS (psychosocial history)? Do we have to do it like with adults? Do have to focus on the complaint or not? well, there you go. It’s just that I don’t really know what to expect at the moment’ (Lamia, session 3)* Excerpt from field notes (session 2): *‘One student interrupts the role play as soon as the somatic history is over, which she has done well. She talks about her discomfort at not knowing how to continue in the HEADS psychosocial interview which is more specific to adolescent health’.*
Uncovering the unknown	
Refers to the perceived added value of having the experience itself but also to how the training helped students observe or practice some interviewing techniques themselves.	*‘It defused something in relation to adolescents. As I said before, it was the first time that I talked to an adolescent being myself out of adolescence. And it put me more at ease.’ (Dimitri, session 2)* *‘I think it’s important to keep this idea that the HEAADSSS interview must be flexible, that it’s not fixed, that it can be modulated and that each adolescent will react differently. And to also have in mind this notion of how to set a framework, […] that it often helps the good progress of the interview … if we explain again and again that it is confidential’ (Marie, session 2)*
3. PROFESSIONAL DISTANCE	
Observing the relational closeness with adolescents	
Refers to how students feel close to and even identify with adolescents either on an emotional level, for example by projecting themselves into their own adolescent experience, or on a social level in terms of age proximity and sharing common interests or activities	*‘So, at one point, it was up to one of my friends to interview him (the adolescent) and I know that my friend likes to smoke joints and so with all my friends, we were laughing about the situation. You know to watch him playing the doctor and giving advice to someone who has problems with cannabis’ (Dimitri, session 2)*
Adjusting the professional distance	
Refers to how students manage distance from their own adolescent experience in the assessment and management of adolescent patients and to how students deal with the small age difference and social proximity in the professional doctor-patient relationship	*‘I used to play at video games, and so did all of my friends. And finally, we ended up at the university, so it doesn’t have a huge impact. It’s cool to play at video games.’ (Lauriane, session 4)*. Excerpt from field notes (session 5): ‘*The simulated patient provides feedback to the student about some smiles during the consultation and her somewhat “cool” attitude that bothered her a bit and that she felt was out of step with the case. The student said she wanted to joke around a bit to lighten the atmosphere. The teacher takes this opportunity to provide some insights about the age difference issue and the need to not act like the adolescent’s buddy by citing a study on this topic. A student then asks for confirmation that one should not adopt such a “cool” attitude.’*

## References

[1] Haller DM, Sanci LA, Patton GC, et al. Toward youth friendly services: a survey of young people in primary care. J Gen Intern Med. 2007 Jun;22(6):775–11. 17380370 10.1007/s11606-007-0177-5 PMC2219862

[2] Gore FM, Bloem PJ, Patton GC, et al. Global burden of disease in young people aged 10–24 years: a systematic analysis. Lancet. 2011 Jun 18;377(9783):2093–2102. 21652063 10.1016/S0140-6736(11)60512-6

[3] Churchill R, Allen J, Denman S, et al. Do the attitudes and beliefs of young teenagers towards general practice influence actual consultation behaviour? Br J Gen Pract. 2000 Dec;50(461):953–957. 11224965 PMC1313880

[4] Donovan C, Mellanby AR, Jacobson LD, et al. Teenagers’ views on the general practice consultation and provision of contraception. The adolescent working group. Br j general practice :j Royal College General Practitioners. 1997 Nov;47(424):715–718. PMC1409956 9519517

[5] Ford CA, Millstein SG, Halpern-Felsher BL, et al. Influence of physician confidentiality assurances on adolescents’ willingness to disclose information and seek future health care. A randomized controlled trial. Jama. 1997 Sep 24;278(12):1029–1034. 9307357

[6] Ambresin A-E, Bennett K, Patton GC, et al. Assessment of youth-friendly health care: a systematic review of indicators drawn from young people’s perspectives. J Adolesc Health. 2013 Jun;52(6):670–681. 23701887 10.1016/j.jadohealth.2012.12.014

[7] Feddock CA, Hoellein AR, Griffith CH, et al. Enhancing knowledge and clinical skills through an adolescent medicine workshop. Arch Pediatr Adolesc Med. 2009;163(3):256–260. 19255394 10.1001/archpediatrics.2008.559

[8] Fallucco EM, James L, Smotherman C, et al. Impact of experiential training with standardized patients on screening and diagnosis of adolescent depression in primary care. J Adolesc Health. 2019 Jul 1;65(1):57–62. 30879884 10.1016/j.jadohealth.2018.12.022

[9] Fallucco EM, Hanson MD, Glowinski AL. Teaching pediatric residents to assess adolescent suicide risk with a standardized patient module. Pediatrics. 2010;125(5):953–959. 20385649 10.1542/peds.2009-2135

[10] Fallucco EM, Conlon MK, Gale G, et al. Use of a standardized patient paradigm to enhance proficiency in risk assessment for adolescent depression and suicide. J Adolesc Health. 2012;51(1):66–72. 22727079 10.1016/j.jadohealth.2011.12.026

[11] Hardoff D, Schonmann S. Training physicians in communication skills with adolescents using teenage actors as simulated patients. Med Educ. 2001;35(3):206–210. 11260441 10.1046/j.1365-2923.2001.00764.x

[12] Brown R, Doonan S, Shellenberger S. Using children as simulated patients in communication training for residents and medical students: a pilot program. Acad Med. 2005;80(12):1114–1120. 16306284 10.1097/00001888-200512000-00010

[13] Bokken L, Van Dalen J, Scherpbier A, et al. Lessons learned from an adolescent simulated patient educational program: five years of experience. Med Teach. 2009;31(7):605–612. 18937136 10.1080/01421590802208891

[14] Bokken L, van Dalen J, Rethans JJ. The case of “Miss Jacobs”: adolescent simulated patients and the quality of their role playing, feedback, and personal impact. Simul Healthc. 2010;5(6):315–319. 21330815 10.1097/SIH.0b013e3181ddcd71

[15] Blake KD, Gusella J, Greaven S, et al. The risks and benefits of being a young female adolescent standardised patient. Med Educ. 2006;40(1):26–35. 16441320 10.1111/j.1365-2929.2005.02343.x

[16] Hanson M, Tiberius R, Hodges B, et al. Adolescent standardized patients: method of selection and assessment of benefits and risks. Teach Learn Med. 2002;14(2):104–113. 12058545 10.1207/S15328015TLM1402_07

[17] Hanson M, Tiberius R, Hodges B, et al. Implications of suicide contagion for the selection of adolescent standardized patients. Acad Med. 2002;77(10 Suppl):S100–2. 12377718 10.1097/00001888-200210001-00031

[18] Hanson MD, Niec A, Pietrantonio AM, et al. Effects associated with adolescent standardized patient simulation of depression and suicidal ideation. Acad Med. 2007;82(Suppl):S61–4. 17895693 10.1097/ACM.0b013e31813ffedd

[19] Silverman J, Kurtz SM, Draper J, et al. Skills for communicating with patients. Oxford UK: Radcliffe Pub; 2005.

[20] Berkhof M, van Rijssen HJ, Schellart AJM, et al. Effective training strategies for teaching communication skills to physicians: an overview of systematic reviews. Patient Educ Couns. 2011 Aug 1;84(2):152–162. 20673620 10.1016/j.pec.2010.06.010

[21] Rees C, Sheard C, McPherson A. Medical students’ views and experiences of methods of teaching and learning communication skills. Patient Educ Couns. 2004 Jul;54(1):119–121. 15210269 10.1016/S0738-3991(03)00196-4

[22] Bokken L, Rethans -J-J, Van Heurn L, van der Vleuten C. et al. Students’ views on the use of real patients and simulated patients in undergraduate medical education. Acad Med. 2009 Jul;84(7):958–963. 19550197 10.1097/ACM.0b013e3181a814a3

[23] Michaud P-A, Jansen D, Schrier L, et al. An exploratory survey on the state of training in adolescent medicine and health in 36 European countries. Eur J Pediatr. 2019;178(10):1559. 31463767 10.1007/s00431-019-03445-1 PMC6733827

[24] Glaser B, Strauss A. The discovery of grounded theory: strategies for qualitative research. Chicago: Aldine; 1967.

[25] Watling CJ, Lingard L. Grounded theory in medical education research: AMEE Guide No. 70. Med Teach. 2012;34(10):850–861. 22913519 10.3109/0142159X.2012.704439

[26] Starks H, Trinidad SB. Choose your method: a comparison of phenomenology, discourse analysis, and grounded theory. Qual Health Res. 2007;17(10):1372–1380. 18000076 10.1177/1049732307307031

[27] Kennedy TJT, Lingard LA. Making sense of grounded theory in medical education. Med Educ. 2006;40(2):101–108. 16451236 10.1111/j.1365-2929.2005.02378.x

[28] Charmaz K. Constructing grounded theory. Thousand Oaks: SAGE Publications Ltd; 2014.

[29] Creswell JW Educational research: planning, conducting, and evaluating quantitative and qualitative research [Internet]. Thousand Oaks: SAGE Publications Ltd; 2012. Available from: https://books.google.ch/books?id=1bk3YgEACAAJ

[30] Saldana J. The coding manual for qualitative researchers. Thousand Oaks: SAGE Publications Ltd; 2012.

[31] Bryant A, Charmaz K. The Sage handbook of grounded theory. Thousand Oaks: SAGE Publications Ltd; 2007.

[32] Fortenberry JD, Kaplan DW, Hill RF. Physicians‘ values and experience during adolescence. Journal of Adolescent Health Care. 1988;9(1):46–51. 3335470 10.1016/0197-0070(88)90017-4

[33] Upadhya KK, Trent ME, Ellen JM. Impact of individual values on adherence to emergency contraception practice guidelines among pediatric residents: implications for training. Arch Pediatr Adolesc Med. 2009 Oct 5;163(10):944–948. 19805714 10.1001/archpediatrics.2009.160 PMC4332886

[34] Salmon P, Young B. The validity of education and guidance for clinical communication in cancer care: evidence-based practice will depend on practice-based evidence. Patient Educ Couns. 2013 Feb 1;90(2):193–199. 22632737 10.1016/j.pec.2012.04.010

[35] Ruiz-Moral R, Gracia de Leonardo C, Caballero MF, et al. Medical students’ perceptions towards learning communication skills: a qualitative study following the 2-year training programme. Int J Med Educ. 2019 May;3(10):90–97. 10.5116/ijme.5cbd.7e96 PMC6766390 31055522

[36] Graves CE, Bridge MD, Nyhuis AW. Residents‘ perception of their skill levels in the clinical management of adolescent health problems. J adol health care : official publ Society Adol Med. 1987 Sep;8(5): 413–418. 10.1016/0197-0070(87)90229-4 3667394

[37] Woods JL, Pasold TL, Boateng BA, et al. Medical student self-efficacy, knowledge and communication in adolescent medicine. Int J Med Educ. 2014;5:165–172. 25341226 10.5116/ijme.53d3.7b30 PMC4212411

[38] Whitt R, Toussaint G, Bruce Binder S, et al. Strengthening student communication through pediatric simulated patient encounters. J Educ Eval Health Prof. 2014 Aug 17;11:21 10.3352/jeehp.2014.11.21 PMC4309937 25112449

[39] Ammentorp J, Sabroe S, Kofoed P-E, et al. The effect of training in communication skills on medical doctors’ and nurses’ self-efficacy. Patient Education and Counseling. 2007;66(3):270–277. 17337337 10.1016/j.pec.2006.12.012

[40] Ozer EM, Adams SH, Gardner LR, et al. Provider self-efficacy and the screening of adolescents for risky health behaviors. J Adolesc Health. 2004;35(2):101–107. 15261638 10.1016/j.jadohealth.2003.09.016

[41] Britto MT, Slap GB, DeVellis RF, et al. Specialists understanding of the health care preferences of chronically ill adolescents. J Adolesc Health. 2007;40(4):334–341. 17367726 10.1016/j.jadohealth.2006.10.020

[42] Peterson WE, Sword W, Charles C, et al. Adolescents‘ perceptions of inpatient postpartum nursing care. Qual Health Res. 2007 Feb;17(2):201–212. 17220391 10.1177/1049732306297414

[43] Halpern J. Empathy and patient–physician conflicts. Journal of General Internal Medicine. 2007 May 1;22(5):696–700. 17443382 10.1007/s11606-006-0102-3 PMC1852904

[44] Halpern J. What is clinical empathy? J Gen Intern Med. 2003 Aug;18(8):670–674. 12911651 10.1046/j.1525-1497.2003.21017.x PMC1494899

[45] Yardley S, Teunissen PW, Dornan T. Experiential learning: AMEE Guide No. 63. 63. Medical Teacher 2012 Feb 1;34(2):e102–15. 22289008 10.3109/0142159X.2012.650741

[46] Sandars J. The use of reflection in medical education: AMEE Guide No. 44. 44. Medical Teacher 2009 Jan 1;31(8):685–695. 19811204 10.1080/01421590903050374

[47] van Houten-Schat MA, Berkhout JJ, van Dijk N, et al. Self-regulated learning in the clinical context: a systematic review. Med Educ. 2018;52(10):1008–1015. 29943415 10.1111/medu.13615 PMC6175376

[48] Edmunds S, Brown G. Effective small group learning: AMEE Guide No. 48. 48. Medical Teacher 2010 Sep;32(9):715–726. 20795801 10.3109/0142159X.2010.505454

[49] Cordovani L, Cordovani D. A literature review on observational learning for medical motor skills and anesthesia teaching. Advances in Health Sciences Education. 2016 Dec 1;21(5):1113–1121. 26506843 10.1007/s10459-015-9646-5

[50] Rizzolatti G, Craighero CL. THE MIRROR-NEURON SYSTEM. Annu Rev Neurosci. 2004;27(1):169–192. 15217330 10.1146/annurev.neuro.27.070203.144230

[51] Jonsen K, Jehn KA. Using triangulation to validate themes in qualitative studies. Qualitative Research in Organizations and Management: An International Journal. 2009 Aug 21;4(2):123–150.

